# Antimicrobial and Biofilm Inhibition Activity of Novel Biobased Quaternary Ammonium Salts

**DOI:** 10.1021/acsomega.5c06267

**Published:** 2025-08-21

**Authors:** Kateřina Sasínová, Markéta Berčíková, Blanka Vrchotová, Iveta Hrádková, Jan Šmidrkal, Katsiaryna Kalenchak

**Affiliations:** University of Chemistry and Technology, Prague 166 28, Czech Republic

## Abstract

The growing threat of antibiotic-resistant bacteria continues to be one of the biggest challenges facing public health. As a result, there is an increasing focus on developing new substances with both antimicrobial and biofilm inhibition activities. One such group of compounds is surfactants, particularly quaternary ammonium salts (QASs), which are commonly used as disinfectants in healthcare. In this study, a three-step synthesis was used to prepare a range of QASs, including quaternary esters, hydroxyamides, and dihydroxyamides with alkyl chains of 12–18 carbon atoms. First, the initial step of the synthesis was optimized by testing various catalysts, with CH_3_OK showing the highest efficiency and proving to be the most suitable choice for further development. Then, the antimicrobial activity was tested against *Staphylococcus aureus*, *Escherichia coli*, *Pseudomonas aeruginosa*, *Candida albicans*, and *Aspergillus brasiliensis*, while biofilm inhibition activity was evaluated only for the bacterial strains (*S. aureus*, *E. coli*, and *P. aeruginosa*). The results were compared with those obtained for benzyldimethyldodecylammonium chloride (BDMDAC), which is a commonly used disinfectant. QASs derived from myristic and palmitic acids showed the highest antimicrobial and biofilm inhibition activities, often higher than BDMDAC. Interestingly, some compounds reached maximum biofilm inhibition activity at the lowest concentration tested—particularly stearic acid quaternary hydroxyamide and stearic acid quaternary dihydroxyamide, which reached minimum biofilm inhibitory concentration (MBIC) values as low as 0.016 mmol L^–1^. Compounds derived from myristic acid showed higher antimicrobial activity compared with BDMDAC, while both myristic- and palmitic-acid–based compounds demonstrated superior biofilm inhibition activity. These findings highlight the potential of myristic- and palmitic-acid–based QASs as promising candidates for next-generation disinfectant formulations, particularly in applications where strong biofilm inhibition activity is essential.

## Graphical abstract

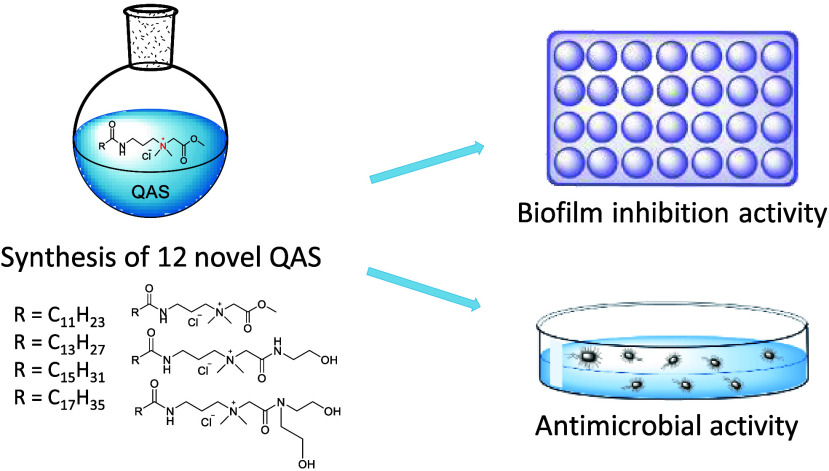

## 1 Introduction

The growing problem of antibiotic resistance among pathogenic bacteria poses a serious threat to public health and challenges current treatment and infection control strategies. As a result, there is an urgent need to develop new disinfectants with broad-spectrum antimicrobial and biofilm inhibition activities to help prevent the spread of resistant strains and address the serious issue of biofilm-associated infections.^1,2^ One group of compounds that has demonstrated strong potential is quaternary ammonium salts (QASs), known for their potent activity against a wide range of microorganisms. With increasing concern over environmental impact and sustainability, current research is focusing on the development of QASs derived from renewable plant-based feedstocks. A promising approach involves the use of natural oils, such as coconut fat, palm kernel oil, and palm oil. These oils are widely used in the oleochemical industry, particularly in the production of surfactants and detergents, due to their favorable fatty acid composition and availability. Coconut fat and palm kernel oil are especially rich in medium-chain-saturated fatty acids, including lauric acid (46–51%), myristic acid (15–19%), and palmitic acid (7–10%), while palm oil predominantly contains palmitic acid (∼39.8%) and stearic acid (∼4.4%).^3–5^ These fatty acids are highly suitable for incorporation into QAS structures, which consist of a central, positively charged nitrogen atom bonded to four organic groups, typically alkyl chains or aromatic moieties, and a counterion, most commonly chloride or bromide.^6^

The positively charged head groups of QASs play a key role in their antimicrobial activity. They interact electrostatically with negatively charged components of the cytoplasmic membrane, such as phospholipids, leading to adsorption onto and penetration through the cell wall. This disrupts membrane organization and causes leakage of intracellular contents (e.g., potassium ions, phosphate, amino acids, proteins, and DNA), ultimately triggering autolysis. At higher concentrations, QASs can also solubilize membrane lipids, further contributing to membrane collapse.^6–12^

Gram-negative bacteria are generally less sensitive to antimicrobials than Gram-positive bacteria, mainly due to differences in the cell wall structure. While the cell wall of Gram-positive bacteria consists of a thick layer of peptidoglycan, allowing easier penetration of substances into the cell, Gram-negative bacteria also possess an outer membrane. This membrane is composed of lipids, lipopolysaccharides, and proteins, forming an additional barrier that QASs must first pass, typically by disrupting or lysing this layer, before they can interact with the inner membrane and intracellular components.^11,13,14^ Therefore, QASs tend to be more effective against Gram-positive bacteria, which lack the outer membrane found in Gram-negative species.

Another possible explanation for the increased resistance of Gram-negative bacteria is the presence of multiple resistance mechanisms, such as efflux pumps, which actively transport antibiotics and other antimicrobials out of bacterial cells, reducing their intracellular concentration.^1,2^

Biofilm formation represents another major resistance mechanism. A biofilm is a complex, structured community of microorganisms embedded in a self-produced extracellular polymeric substance (EPS), which consists of polysaccharides, proteins, lipids, and extracellular DNA (eDNA). The EPS provides structural integrity, protection, and increased resistance to antibiotics, while eDNA (released through cell lysis or secretion) plays a crucial role in biofilm stability, gene exchange, and antimicrobial resistance. Biofilm formation is controlled by several interconnected signaling pathways, including quorum sensing (QS), which helps bacteria coordinate their behavior by releasing and detecting signaling molecules called autoinducers.^1,15,16^

The mechanism of biofilm eradication by QASs involves reducing the surface tension and disrupting bacterial adhesion. Thanks to their amphipathic properties, QASs bind to the hydrophobic parts of bacterial membranes, preventing attachment to surfaces and the formation of biofilms.^17^

The antimicrobial and biofilm inhibition activities of QASs have been shown to depend significantly on the length of their hydrocarbon chain. Several studies suggest a clear correlation between the chain length and antimicrobial activity, with optimal results often associated with specific chain lengths. For example, Obłąk reported the highest activity for compounds containing 12 and 14 carbon atoms. In contrast, Paluch observed the strongest effects against planktonic bacteria for compounds with 14 and 16 carbon atoms. Gozzelino also observed peak activity for 16 carbon compounds against planktonic forms, while Kula reported that compounds with 16 carbon atoms were the most effective against both planktonic forms and biofilms.^18–21^

While alkyl chain length is recognized as a key structural parameter influencing the antimicrobial efficacy of QASs, previous studies have reported inconsistent findings, particularly regarding the optimal chain length and its role in biofilm inhibition. To address this, the present study aimed to synthesize a new series of QASs to further investigate how the hydrocarbon chain length influences both antimicrobial and biofilm inhibition activities. Understanding this relationship is particularly important, given the growing problem of antimicrobial resistance. Such compounds could offer valuable applications in both industrial and clinical disinfections, especially against planktonic bacteria and biofilms. At the same time, the use of renewable starting materials supports the development of QASs that offer both effective biological activity and enhanced environmental sustainability.

## 2 Experimental Procedure

### 2.1 Materials

3-(Dimethylamino)-1-propylamine (≥99.0%), acetonitrile (99.9%), benzyldimethyldodecylammonium chloride (BDMDAC) (≥99.0%), diethanolamine (≥98.0%), methyl chloroacetate (99.0%), methyl laurate (99.5%, acid value (AV) 0.77 mg KOH g^–1^), methyl myristate (99.6%, AV 0.53 mg KOH g^–1^), methyl palmitate (98.7%, AV 0.21 mg KOH g^–1^), methyl stearate (98.2%, AV 1.20 mg KOH g^–1^), monoethanolamine (99.0%), lithium hydroxide (≥99.0%), lithium methoxide (10% solution in methanol), potassium hydroxide (45% solution in water), potassium methoxide (25% solution in methanol), rubidium hydroxide (50% solution in water), sodium hydroxide (50% solution in water), and sodium methoxide (25% solution in methanol) were purchased from Merck. Acetone (99.5%), ammonia aqueous solution (24.0%), dichloromethane (p.a.), glycerol (p.a.), isopropanol (p.a.), methanol (p.a.), and *n*-hexane (p.a.) were purchased from Penta. Cesium hydroxide (99.9%) was purchased from Alfa Aesar. Tween 80 was purchased from HiMedia.

Microbiological media: Nutrient Broth, Tryptic Soy Agar, and Malt Extract Agar were purchased from Oxoid, Sabouraud Dextrose Agar (VWR), and Potato Dextrose Agar (Merck), respectively. Phosphate buffer (PBS) was prepared according to CHS Protocols.^22^

### 2.2 Synthesis of Quaternary Ammonium Salts

The synthesis of quaternary ammonium salts (QASs) derived from lauric, myristic, palmitic, and stearic acid was carried out through a three-step synthesis (Figure 1). For clarity, only the synthesis of QASs derived from lauric acid is described below. The remaining compounds were prepared using a similar procedure, with differences noted in Tables 1–4.

**Table 1:** Reaction Conditions for Synthesis of Aminoamide^a^

compound	methyl ester [mol]	3-DMAPA [mol]	CH_3_ONa [mol]	glycerol [g]	*t* [h]
1A	0.05	0.052	0.002	-	4
1B	0.05	0.052	0.002	-	4
1C	0.05	0.052	0.002	-	4
1D	0.05	0.052	0.004	1	40

a 1, Aminoamide; A, lauric acid; B, myristic acid; C, palmitic acid; D, stearic acid; 3-DMAPA, 3-(dimethylamino)-1-propylamine.

**Table 2:** Reaction Conditions for Synthesis of Quaternary Ester^a^

compound	aminoamide [mol]	methyl chloroacetate [mol]	methanol [mL]
2A	0.023	0.025	23
2B	0.021	0.023	23
2C	0.02	0.022	24
2D	0.024	0.026	31

a 2, Quaternary ester; A, lauric acid; B, myristic acid; C, palmitic acid; D, stearic acid.

**Table 3:** Reaction Conditions for the Synthesis of Quaternary Hydroxyamide^a^

compound	quaternary ester [mol]	monoethanolamine [mol]	acetonitrile [mL]
3A	0.011	0.011	15
3B	0.01	0.01	14
3C	0.009	0.01	15
3D	0.011	0.012	17

a 3, Quaternary hydroxyamide; A, lauric acid; B, myristic acid; C, palmitic acid; D, stearic acid.

**Table 4:** Reaction Conditions for the Synthesis of Quaternary Dihydroxyamide^a^

compound	auaternary ester [mol]	diethanolamine [mol]	acetonitrile [mL]
4A	0.011	0.011	15
4B	0.01	0.01	14
4C	0.009	0.01	15
4D	0.011	0.012	17

a 4, Quaternary dihydroxyamide; A, lauric acid; B, myristic acid; C, palmitic acid; D, stearic acid.

**Figure 1:**
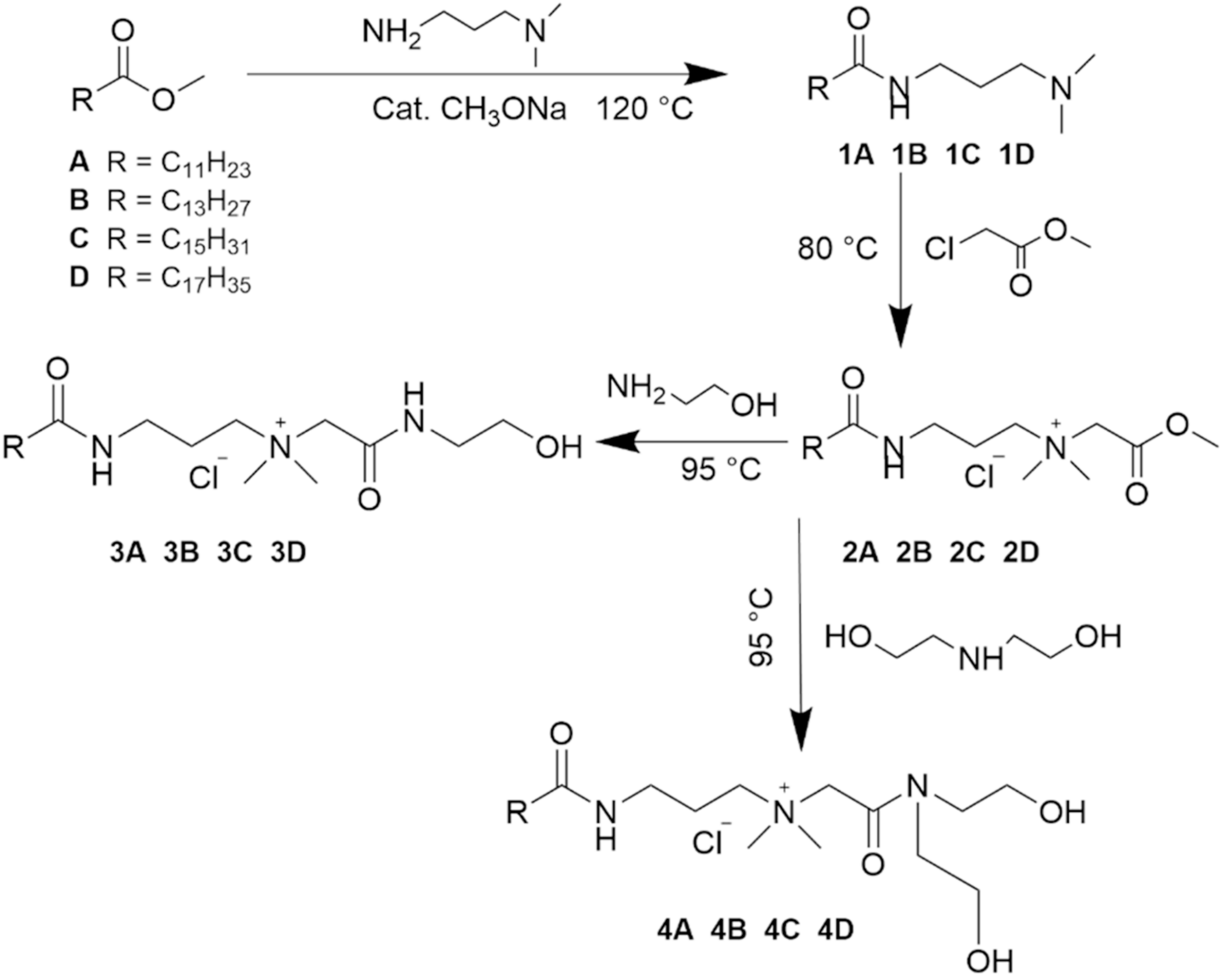
Synthesis of quaternary ammonium salts.

In the first step, a fatty acid methyl ester reacted with 3-(dimethylamino)­propylamine (3-DMAPA) to form an aminoamide (1A–1D). Subsequently, the aminoamide (1A–1D) was quaternized with methyl chloroacetate to yield a quaternary ester (2A–2D). The final step of the synthesis was divided into two reactions. In the first reaction, aminolysis with monoethanolamine was carried out, resulting in the formation of a quaternary hydroxyamide (3A–3D). In the second reaction, aminolysis with diethanolamine was performed, leading to the production of a quaternary dihydroxyamide (4A–4D).

#### 2.2.1 Synthesis of Aminoamide

In the first step of the synthesis, a reaction occurs between the fatty acid methyl ester and 3-DMAPA in a molar ratio of 1:1.08 (methyl ester/3-DMAPA). The reaction conditions for all methyl esters are given in Table 1.

First, 5.314 g (0.052 mol) of 3-DMAPA was added to a three-necked flask. Then, 0.432 g (0.002 mol) of a 25% solution of sodium methoxide in methanol, used as the catalyst, was added in excess to react with free fatty acids to form corresponding fatty acid salts. Subsequently, 10.72 g (0.05 mol) of methyl laurate was added to the flask. The reaction proceeded with constant stirring at 200 rpm for 4 h at 120 °C in an argon atmosphere. The reaction was monitored by using thin-layer chromatography. Upon completion of the reaction, 70 mL of dichloromethane was added to the mixture, and the catalyst was removed by filtration. Dichloromethane was subsequently removed with a vacuum evaporator (IKA RV 10 digital). The reaction mixture (in a 1:1 ratio with the solvent) was crystallized from 15 mL of methanol at 5 °C for 24 h. Dimethylaminopropylamide of lauric acid 1A (6.54 g, 46% yield) was obtained as white crystals. This procedure was the same for all other compounds derived from the remaining fatty acids except for stearic acid. In this case, the reaction was slow under these conditions. For this reason, glycerol was added to the mixture, the amount of catalyst was increased, and the time of reaction was prolonged.

The physical properties of the synthesized compounds are listed in Table 5. The corresponding ^1^H NMR spectra were then measured and are presented below:

**Table 5:** Characterization of Synthesized Compounds^a^

compound	R	*M* [g moL^–1^]	yield [%]	melting point [°C]	*R*_f_	CMC [mol L^–1^]
1A	C_11_H_23_	284.49	46	31–32	0.44	-
1B	C_13_H_27_	312.54	41	45–47	0.58	-
1C	C_15_H_31_	340.60	40	53–55	0.54	-
1D	C_17_H_35_	368.65	48	65–67	0.5	-
2A	C_11_H_23_	393.01	95	47–79	0.19	1.1·10^–3^
2B	C_13_H_27_	421.04	97	61–63	0.32	1.04·10^–3^
2C	C_15_H_31_	449.12	96	64–66	0.29	1.17·10^–4^
2D	C_17_H_35_	477.17	95	65–68	0.27	5.28·10^–5^
3A	C_11_H_23_	422.05	86	73−74	0.03	3.76·10^–3^
3B	C_13_H_27_	450.11	98	71–74	0.03	9.66·10^–4^
3C	C_15_H_31_	478.16	92	76–78	0.03	8.47·10^–5^
3D	C_17_H_35_	506.21	73	81–84	0.03	5.69·10^–5^
4A	C_11_H_23_	466.10	85	46−47	0.03	1.96·10^–3^
4B	C_13_H_27_	494.16	66	59–60	0.03	3.95·10^–4^
4C	C_15_H_31_	522.21	98	59–60	0.03	3.75·10^–5^
4D	C_17_H_35_	550.27	79	56–58	0.03	3.69·10^–5^

a 1 – Aminoamide; 2 – quaternary ester; 3 – quaternary hydrodroxyamide 4 – quaternary dihydroxyamide; A - lauric acid; B - myristic acid; C - palmitic acid; D - stearic acid.

##### 2.2.1.1 *N*-(3-(Dimethylamino)­Propyl)­Dodecanamide (**1A**)

1H NMR (500 MHz, CDCl3): δ 0.87 (t, 3H, Ca–H3), 1.28 (m, 8H, Cb–H2), 1.64 (p, 2H, Cc–H2), 1.64 (p, 2H, Cd–H2), 2.14 (t, 2H, Ce–H2), 2.22 (s, 6H, Cf–H3), 2.38 (t, 2H, Cg–H2), 3.33 (dt, 2H, Ch–H2), 6.89 (s, 1H, Ci–H).

##### 2.2.1.2 *N*-(3-(Dimethylamino)­Propyl)­Tetradecanamide (**1B**)

1H NMR (500 MHz, CDCl3): δ 0.87 (t, 3H, Ca–H3), 1.28 (m, 10H, Cb–H2), 1.64 (p, 2H, Cc–H2), 1.64 (p, 2H, Cd–H2), 2.15 (t, 2H, Ce–H2), 2.22 (s, 6H, Cf–H3), 2.38 (t, 2H, Cg–H2), 3.34 (dt, 2H, Ch–H2), 6.88 (s, 1H, Ci–H).

##### 2.2.1.3 *N*-(3-(Dimethylamino)­Propyl)­Hexadecanamide (**1C**)

1H NMR (500 MHz, CDCl3): δ 0.88 (t, 3H, Ca–H3), 1.28 (m, 12H, Cb–H2), 1.63 (p, 2H, Cc–H2), 1.65 (p, 2H, Cd–H2), 2.15 (t, 2H, Ce–H2), 2.22 (s, 6H, Cf–H3), 2.37 (t, 2H, Cg–H2), 3.33 (dt, 2H, Ch–H2), 6.89 (s, 1H, Ci–H).

##### 2.2.1.4 *N*-(3-(Dimethylamino)­Propyl)­Oktadecanamide (**1D**)

1H NMR (500 MHz, CDCl3): δ 0.89 (t, 3H, Ca–H3), 1.27 (m, 28H, Cb–H2), 1.62 (m, 2H, Cc–H2), 1.65 (p, 2H, Cd–H2), 2.14 (t, 2H, Ce–H2), 2.23 (s, 6H, Cf–H3), 2.37 (t, 2H, Cg–H2), 3.33 (dt, 2H, Ch–H2), 6.90 (s, 1H, NH).

#### 2.2.2 Synthesis of Quaternary Ester

In the second step of the synthesis, *N*-(*3*-(*dimethylamino*)*propyl*)­alkylamide (1) of the respective fatty acid was reacted with methyl chloroacetate in a molar ratio of 1:1.1 (dimethylaminopropylamide/methyl chloroacetate). Reaction conditions for all compounds are listed in Table 2.

In a two-neck flask, 6.54 g (0.023 mol) of *N*-(*3*-(*dimethylamino*)*propyl*)­dodecanamide (1A) and 2.71 g (0.025 mol) of methyl chloroacetate were weighed. Subsequently, 23 mL of methanol was added to the mixture. The reaction was conducted under constant stirring at 200 rpm and refluxed at 80 °C for 14 h. The reaction was monitored using thin-layer chromatography. After the reaction was completed, the methanol was evaporated using a rotary evaporator (IKA RV 10 digital). The reaction mixture was crystallized from 20 mL of acetone at −20 °C. Quaternary ester of lauric acid 2A (8.6 g, 95% yield) was obtained as white crystals.

The physical properties of the synthesized compounds are listed in Table 5. The corresponding ^1^H NMR spectra were then measured and are presented below:

##### 2.2.2.1 2-Methoxy-*N*,*N*-Dimethyl-2-Oxo-*N*-(2-Dodecanamidoethyl)­Ethan-1-Aminium Chloride (**2A**)

1H NMR (500 MHz, CDCl3): δ 0.86 (t, 3H, Ca–H3), 1.25 (m, 8H, Cb–H2), 1.59 (p, 2H, Cc–H2), 2.08 (p, 2H, Cd–H2), 2.26 (t, 2H, Ce–H2), 3.35 (t, 2H, Cf–H2), 3.49 (s, 6H, Cg–H3), 3.81 (s, 3H, Ch–H3), 4.05 (dt, 2H, Ci–H2), 4.84 (s, 2H, Cj–H2), 7.84 (s, 1H, Ck–H).

##### 2.2.2.2 2-Methoxy-*N*,*N*-Dimethyl-2-Oxo-*N*-(2-Tetradecanamidoethyl)­Ethan-1-Aminium Chloride (**2B**)

1H NMR (500 MHz, CDCl3): δ 0.86 (t, 3H, Ca–H3), 1.23 (m, 10H, Cb–H2), 1.58 (p, 2H, Cc–H2), 2.08 (p, 2H, Cd–H2), 2.26 (t, 2H, Ce–H2), 3.34 (t, 2H, Cf–H2), 3.49 (s, 6H, Cg–H3), 3.80 (s, 3H, Ch–H3), 4.00 (dt, 2H, Ci–H2), 4.81 (s, 2H, Cj–H2), 7.88 (s, 1H, Ck–H).

##### 2.2.2.3 2-Methoxy-*N*,*N*-Dimethyl-2-Oxo-*N*-(2-Palmitamidoethyl)­Ethan-1-Aminium Chloride (**2C**)

1H NMR (500 MHz, CDCl3): δ 0.85 (t, 3H, Ca–H3), 1.22 (m, 12H, Cb-H2), 1.56 (p, 2H, Cc-H2), 2.07 (p, 2H, Cd–H2), 2.23 (t, 2H, Ce–H2), 3.30 (t, 2H, Cf–H2), 3.47 (s, 6H, Cg-H3), 3.79 (s, 3H, Ch-H3), 3.96 (dt, 2H, Ci-H2), 4.77 (s, 2H, Cj-H2), 7.89 (s, 1H, Ck-H).

##### 2.2.2.4 2-Methoxy-*N*,*N*-Dimethyl-2-Oxo-*N*-(2-Stearamidoethyl)­Ethan-1-Aminium Chloride (**2D**)

1H NMR (500 MHz, CDCl3): δ 0.92 (t, 3H, Ca–H3), 1.30 (m, 14H, Cb–H2), 1.64 (dt, 2H, Cc–H2), 1.80 (dp, 2H, Cd–H2), 2.32 (dt, 2H, Ce–H2), 3.39 (dt, 2H, Cf–H2), 3.53 (s, 6H, Cg–H3), 3.86 (m, 2H, Ci–H2), 4.02 (s, 3H, Ch–H3), 4.85 (s, 2H, Cj–H2), 7.84 (s, 1H, Ck–H).

#### 2.2.3 Synthesis of Quaternary Hydroxyamide

The third step of the synthesis was divided into two separate reactions. In the first one, quaternary ester of the fatty acid (2) reacted with monoethanolamine, while in the second one, quaternary ester of the fatty acid (2) reacted with diethanolamine. Both reactions were conducted in a molar ratio of 1:1.05. Since the procedure for both reactions was identical, it will be described in detail here using the reaction with monoethanolamine as an example. The same methodology was applied in Section 2.2.4. Reaction conditions for all compounds are listed in Table 3.

In a two-neck flask, 4.52 g (0.011 mol) of the lauric acid quaternary ester (2A) and 0.69 g (0.011 mol) of monoethanolamine were mixed with 15 mL of acetonitrile. The reaction was carried out under reflux at 95 °C for 6 h with constant stirring at 200 rpm. After the reaction, acetonitrile was removed by using a rotary evaporator (IKA RV 10 digital). Subsequently, 60 mL of acetone was added to the mixture, and crystallization was carried out (quaternary hydroxyamide/acetone 1:10 w/w) at 6 °C for 24 h (4 g, 86% yield). Crystallization was unsuccessful in separating the quaternary esters (2) from the quaternary hydroxyamides (3). As a result, the quaternary hydroxyamide of lauric acid (3A) was first purified by column chromatography (Silica gel 60; dichloromethane/methanol/ammonia, 8:2:0.05, v/v/v) and then recrystallized from acetone (quaternary hydroxyamide/acetone 1:10 w/w). The yield after column chromatography was 65%, and the final product was obtained as white crystals.

The physical properties of the synthesized compounds are listed in Table 5. The corresponding ^1^H NMR spectra were then measured and are presented below:

##### 2.2.3.1 2-((2-Hydroxyethyl)­Amino)-*N*,*N*-Dimethyl-2-Oxo-*N*-(2-Dodecanamidoethyl)­Ethan-1-Aminium Chloride (**3A**)

1H NMR (500 MHz, CDCl3): δ 0.90 (t, 3H, Ca–H3), 1.28 (m, 8H, Cb–H2), 1.62 (p, 2H, Cc–H2), 2.13 (p, 2H, Cd–H2), 2.25 (t, 2H, Ce–H2), 3.37 (t, 4H, Cf–H2), 3.48 (s, 6H, Cg–H3), 3.73 (dt, 4H, Ch–H2), 4.45 (s, 2H, Ci–H2), 7.61 (s, 1H, Cj–H), 9.03 (s, 1H, Ck–H).

##### 2.2.3.2 2-((2-Hydroxyethyl)­Amino)-*N*,*N*-Dimethyl-2-Oxo-*N*-(2-Tetradecanamidoethyl)­Ethan-1-Aminium Chloride (**3B**)

1H NMR (500 MHz, CDCl3): δ 0.87 (t, 3H, Ca–H3), 1.24 (m, 10H, Cb–H2), 1.58 (p, 2H, Cc–H2), 2.09 (p, 2H, Cd–H2), 2.21 (t, 2H, Ce–H2), 3.32 (t, 4H, Cf–H2), 3.36 (s, 6H, Cg–H3), 3.69 (dt, 4H, Ch–H2), 4.39 (s, 2H, Ci–H2), 7.55 (s, 1H, Cj–H), 8.93 (s, 1H, Ck–H).

##### 2.2.3.3 2-((2-Hydroxyethyl)­Amino)-*N*,*N*-Dimethyl-2-Oxo-*N*-(2-Palmitamidoethyl)­Ethan-1-Aminium Chloride (**3C**)

1H NMR (500 MHz, CDCl3): δ 0.86 (t, 3H, Ca–H3), 1.23 (m, 12H, Cb–H2), 1.57 (p, 2H, Cc–H2), 2.09 (p, 2H, Cd–H2), 2.20 (t, 2H, Ce–H2), 3.32 (t, 4H, Cf–H2), 3.36 (s, 6H, Cg–H3), 3.69 (dt, 4H, Ch–H2), 4.42 (s, 2H, Ci–H2), 7.57 (s, 1H, Cj–H), 9.01 (s, 1H, Ck–H).

##### 2.2.3.4 2-((2-Hydroxyethyl)­Amino)-*N*,*N*-Dimethyl-2-Oxo-*N*-(2-Stearamidoethyl)­Ethan-1-Aminium Chloride (**3D**)

1H NMR (500 MHz, CDCl3): δ 0.91 (t, 3H, Ca–H3), 1.29 (m, 14H, Cb–H2), 1.63 (dt, 2H, Cc–H2), 2.15 (dp, 2H, Cd–H2), 2.26 (dt, 2H, Ce–H2), 3.38 (dt, 4H, Cf–H2), 3.79 (dt, 4H, Ch–H2), 7.57 (s, 1H, Cj–H), 9.22 (s, 1H, Ck–H).

#### 2.2.4 Synthesis of Quaternary Dihydroxyamide

The procedure of this reaction was the same as that in part Section 2.2.3, and reaction conditions for all compounds are listed in Table 4. In a two-neck flask, 4.52 g (0.011 mol) of the lauric acid quaternary ester (2A) and 1.18 g (0.011 mol) of diethanolamine were mixed with 15 mL of acetonitrile. Quaternary dihydroxyamide of lauric acid 4A (4.4 g, 85%) was purified by column chromatography (Silica gel 60; dichloromethane/methanol/ammonia, 8:2:0.05; v/v/v) and subsequently crystallized from acetone (quaternary dihydroxyamide/acetone 1:10 w/w) to yield white crystals.

The physical properties of the synthesized compounds are listed in Table 5. The corresponding ^1^H NMR spectra were then measured and are presented below:

##### 2.2.4.1 2-(Bis­(2-Hydroxyethyl)­amino)-*N*,*N*-Dimethyl-2-Oxo-*N*-(2-Dodecamidoethyl)­Ethan-1-Aminium Chloride (**4A**)

1H NMR (500 MHz, CDCl3): δ 0.88 (t, 3H, Ca–H3), 1.25 (m, 16H, Cb–H2), 1.58 (m, 2H, Cc–H2), 2.07 (m, 2H, Cd–H2), 2.22 (m, 2H, Ce–H2), 3.08 (t, 2H, Cf–H2), 3.29 (s, 2H, Cg–H2), 3.40 (s, 6H, Ch–H3), 3.54 (d, 4H, Ci–H2), 3.76 (s, 6H, Cj–H2), 4.74 (s, 2H, OH), and 7.70 (s, 1H, NH).

##### 2.2.4.2 2-(Bis­(2-Hydroxyethyl)­Amino)-*N*,*N*-Dimethyl-2-Oxo-*N*-(2-Tetradecamidoethyl)­Ethan-1-Aminium Chloride (**4B**)

1H NMR (500 MHz, CDCl3): δ 0.91 (t, 3H, Ca–H3), 1.28 (m, 10H, Cb–H2), 1.61 (dt, 2H, Cc–H2), 2.07 (dp, 2H, Cd–H2), 2.23 (dt, 2H, Ce–H2), 3.33 (s, 2H, Cf–H2), 3.43 (s, 6H, Cg–H3), 3.54 (s, 4H, Ch–H2), 3.60 (s, 2H, Ci–H2), 3.78 (s, 4H, Cj–H2), 3.88 (s, 2H, Ck–H2), 4.80 (s, 2H, OH), 7.74 (s, 1H, NH).

##### 2.2.4.3 2-(Bis­(2-Hydroxyethyl)­Amino)-*N*,*N*-Dimethyl-2-Oxo-*N*-(2-Palmitamidoethyl)­Ethan-1-Aminium Chloride (**4C**)

1H NMR (500 MHz, CDCl3): δ 0.91 (t, 3H, Ca–H3), 1.28 (m, 12H, Cb–H2), 1.61 (dt, 2H, Cc–H2), 2.09 (dp, 2H, Cd–H2), 2.24 (dt, 2H, Ce–H2), 3.33 (s, 2H, Cf–H2), 3.44 (s, 6H, Cg–H3), 3.53 (s, 4H, Ch–H2), 3.60 (s, 2H, Ci–H2), 3.79 (s, 4H, Cj–H2), 3.88 (s, 2H, Ck–H2), 4.82 (s, 2H, OH), 7.69 (s, 1H, NH).

##### 2.2.4.4 2-(Bis­(2-Hydroxyethyl)­Amino)-*N*,*N*-Dimethyl-2-Oxo-*N*-(2-Stearamidoethyl)­Ethan-1-Aminium Chloride (**4D**)

1H NMR (500 MHz, CDCl3): δ 0.91 (t, 3H, Ca–H3), 1.29 (m, 14H, Cb–H2), 1.61 (dt, 2H, Cc–H2), 2.10 (dp, 2H, Cd–H2), 2.24 (dt, 2H, Ce–H2), 3.33 (s, 2H, Cf–H2), 3.43 (s, 6H, Cg–H3), 3.54 (s, 4H, Ch–H2), 3.61 (s, 2H, Ci–H2), 3.79 (s, 4H, Cj–H2), 3.94 (s, 2H, Ck–H2), 4.81 (s, 2H, OH), 7.72 (s, 1H, NH).

#### 2.2.5 Optimization of the First Reaction Step

To enhance the efficiency of the initial reaction step, a series of catalysts was evaluated. Eight distinct catalysts, comprising five hydroxides and three methoxides, were tested: lithium hydroxide (LiOH), sodium hydroxide (NaOH), potassium hydroxide (KOH), rubidium hydroxide (RbOH), cesium hydroxide (CsOH), lithium methoxide (CH_3_OLi), sodium methoxide (CH_3_ONa), and potassium methoxide (CH_3_OK). Methyl laurate was used as the methyl ester for the reactions with the catalysts listed above. First, 10.72 g (0.05 mol) of methyl laurate was mixed with 5.314 g (0.052 mol) of 3-DMAPA in a three-necked bottle and heated to 120 °C. Once the mixture reached 120 °C, the catalyst was added in varying amounts (0, 0.25, 0.5, 1, 2, and 4 mmol). The reaction was carried out under an argon atmosphere with continuous stirring at 200 rpm. Samples were collected at specific time intervals (0, 10, 20, 40, 80, 160, and 320 min) and analyzed using gas chromatography with flame ionization detection (GC-FID).

### 2.3 Methods

#### 2.3.1 Analytical Methods

Melting points were determined on a Kofler block attached to the microscope (Franz Küstner Nachf.KG, Germany). The rate of heating was 4 °C min^–1^.

The purity of the compounds and the monitoring of the reaction were determined by using thin-layer chromatography (TLC). In the stationary phase, silica gel plates F_254_ from Merck were used. Mobile phase was a mixture of isopropanol/ammonia (24%)/water (8:1:1, v/v/v). For visualization, a 5% solution of phosphomolybdic acid in ethanol was applied on the plates and then heated.

To determine the purity of methyl esters, gas chromatography with a flame ionization detector (GC-FID) was performed using an Agilent Technologies 6890 instrument equipped with a Supelco SP-2560 column (100 m × 0.25 mm, stationary phase thickness of 0.2 μm). Helium was used as the carrier gas at a flow rate of 1 mL min^–1^. The flame ionization detector was maintained at 220 °C, with hydrogen at 45 mL min^–1^, air at 450 mL min^–1^, and nitrogen (makeup gas) at 45 mL min^–1^. Samples were injected with an Agilent Technologies 7683 autosampler at an injection temperature of 220 °C, with a 1 μL injection volume and a split ratio of 1:50.

^1^H NMR spectra were measured. The analysis was performed using a Bruker Avance III 500 MHz instrument (frequency for ^1^H nuclei was 500 MHz, sample was dissolved in deuterated chloroform).

The critical micelle concentration (CMC) was determined by using the ring method with a Lauda TE2 tensiometer (Lauda Scientific) equipped with a platinum du Noüy ring as the measuring element. Measurements were conducted at a controlled temperature of 20.0 ± 0.01 °C, and the samples were added using an automatic buret.

#### 2.3.2 Antimicrobial Activity

Antimicrobial activity of the synthesized QAS was tested against *Escherichia coli* CCM 4517, *Pseudomonas aeruginosa* CCM 1961, *Staphylococcus aureus* CCM 4516, *Candida albicans* CCM 8215, and *Aspergillus brasiliensis* CCM 8222. The bacteria were cultured on Tryptic Soy Agar at 37 °C for 24 h, the yeast was cultured on Sabouraud Dextrose Agar at 30 °C for 48 h, and the fungi were cultured on Potato Dextrose Agar at 25 °C for 168 h. The tested compounds were added to broths (nutrient broth for bacteria and malt extract broth for yeast and fungi) at concentrations of 0.5, 0.25, 0.125, 0.06, 0.03, and 0.016 mmol L^–1^. Bacterial and yeast inocula were prepared by suspending colonies in physiological saline (8.5 g of NaCl per 1 L of distilled water). The suspensions were adjusted by measuring absorbance until reaching an optical density (OD) of 0.45–0.55, measured at 650 nm for bacteria and 630 nm for yeast, to standardize the cell concentration. Fungal inoculum was prepared from mature fungal cultures. To release the spores, 10 mL of Tween 80 solution (0.5 g/L) was added to the culture surface. The resulting suspension was then filtered to remove hyphal fragments. The spores were then counted to ensure a final concentration between 1·10^–6^ and 1·10^–7^ CFU mL^–1^. Then, microtiter plate wells were filled with 200 μL of this mixture and inoculated with a 1% (v/v) microbial suspension. Plates were incubated at 37 °C for 24 h (bacteria), 30 °C for 48 h (yeast), and 25 °C for 168 h (fungi). Microbial growth was quantified spectrophotometrically (PowerWave HT, BioTek Instruments Inc.) by measuring optical density at 650 nm for bacteria and 630 nm for yeast. Growth curves were recorded over time for each tested condition. To quantify growth inhibition, the area under the curve (AUC) was calculated for each condition using the trapezoidal rule. The inhibitory index (II) was then determined as a percentage using the following formula.^23^

$$\mathrm{II}=1-\frac{A_{\mathrm{sample}}}{A_{\mathrm{control}}}100$$

*A*_sample_ is the area under the growth curves of the treated sample (24 h for bacteria, 48 h for yeast), *A*_control_ is the area under the growth curves of the untreated sample (24 h for bacteria, 48 h for yeast), and antifungal activity against fungi was assessed visually.

#### 2.3.3 Biofilm Inhibition Activity

Biofilm inhibition activity was tested on bacterial strains as listed in Section 2.3.2, which were cultivated under identical conditions. The tested compounds were added to nutrient broth at the same concentrations used for minimum inhibitory concentration (MIC) testing. Bacterial inocula were prepared following the same procedure as that for MIC testing, with absorbance adjusted to an OD of 0.45–0.55 at 650 nm. Microtiter plate wells were filled with 200 μL of nutrient broth inoculated with 1% (v/v) bacterial culture and incubated at 37 °C for 48 h. After incubation, wells were washed twice with PBS (200 μL) to remove planktonic cells and left at laboratory temperature until fully dried. Biofilms were stained with 0.1% crystal violet solution in distilled water (200 μL) under constant shaking (20 min at 130 rpm) on a laboratory shaker (Heidolph Unimax 1010). Excess dye was then removed, and the wells were washed twice with PBS, dried, and treated with 200 μL of 96% ethanol to solubilize the stain under constant shaking (10 min, 130 rpm). The ethanol solution (100 μL) was transferred by pipette to a clean plate, and absorbance was measured at 580 nm using a spectrophotometer. Results were averaged from four replicates and compared to those of an uninoculated control.

## 3 Results and Discussion

### 3.1 Synthesis of Quaternary Ammonium Salts

Quaternary ammonium salts derived from various fatty acid methyl esters were synthesized as previously described in Section 2.2. Characterization of the synthesized compounds is summarized in Table 5. The yield of the first step of the synthesis ranged between 40 and 48%. Product losses occurred during the removal of the catalyst from the mixture. The yield of the second step of the synthesis was between 95 and 97%. The yield of the third step of the reaction ranged between 73 and 98% for quaternary hydroxyamides and between 66 and 98% for quaternary dihydroxyamides.

As for the melting point, it was confirmed that increasing the length of the hydrocarbon chain also increases the melting point.

The critical micelle concentration (Table 5) data confirmed that as the hydrocarbon chain length increases, the CMC decreases. This phenomenon is attributed to the decreasing hydrophilicity of the compounds, which leads to lower water solubility and consequent micelle formation at lower concentrations. When comparing the effect of the hydroxyl group in quaternary hydroxyamides and quaternary dihydroxyamides, it was observed that the presence of two hydroxyl groups slightly decreased the CMC value, which is also supported by the findings of Mirgorodskaya et al.^24^

### 3.2 Optimization of the First Reaction Step

To optimize the first reaction step, three methoxides (CH_3_OLi, CH_3_ONa, and CH3OK) and five hydroxides (LiOH, NaOH, KOH, RbOH, and CsOH) were evaluated as catalysts. Among the methoxides, CH_3_OK demonstrated the highest efficiency (Figure 2A). The influence of catalyst amount on the reaction yield follows the expected trend at higher concentrations (1–4 mmol for CH_3_OK (Figure 2B) and 2–4 mmol for CH_3_ONa (Figure 2C)). Here, the increasing amount of catalyst accelerates the reaction without affecting the final equilibrium yield of ∼95%. Interestingly, at a catalyst concentration of 4 mmol, the equilibrium yield (∼95%) was reached within 20 min using CH_3_ONa and within 40 min using CH_3_OK. In contrast, when 2 mmol of catalyst was applied, the same yield was achieved for both catalysts after 160 min. In contrast, at lower concentrations (below 0.5 mmol of CH_3_OK and below 1 mmol of CH_3_ONa), the reaction reached a much lower plateau (∼62%) despite running for an extended period. This suggests that the true thermodynamic equilibrium was likely not reached in this case due to insufficient base concentration. Additionally, potential catalyst deactivation (e.g., reaction with impurities or water) and mass transfer limitations could contribute to the lower observed yield. In contrast to the other tested catalysts, CH_3_OLi proved to be unsuitable, as even after a prolonged reaction time (200 min) and increased catalyst loading (4 mmol), the yield did not exceed 80% (Figure 2D).

**Figure 2:**
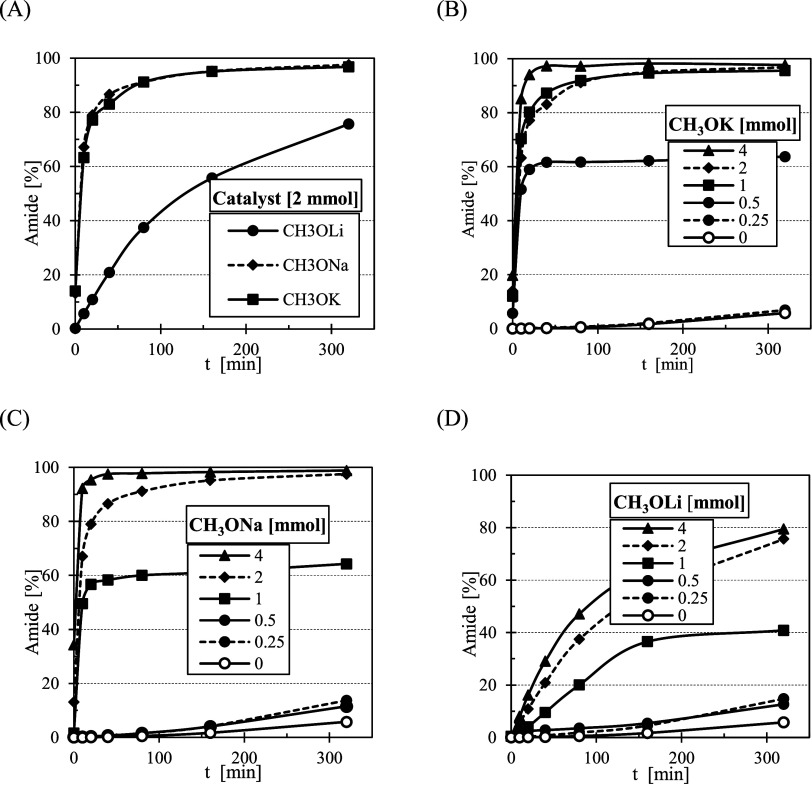
Effect of the catalyst on the reaction: (A) all methoxides in 2 mmol, (B) potassium methoxide, (C) sodium methoxide, and (D) lithium methoxide.

The differences in the catalytic efficiency among the hydroxides were less expressive (Figure 3). CsOH exhibited the highest activity among them; however, even with 2 mmol of catalyst, the reaction did not reach equilibrium within 300 min, and the product yield remained below 30%.

**Figure 3:**
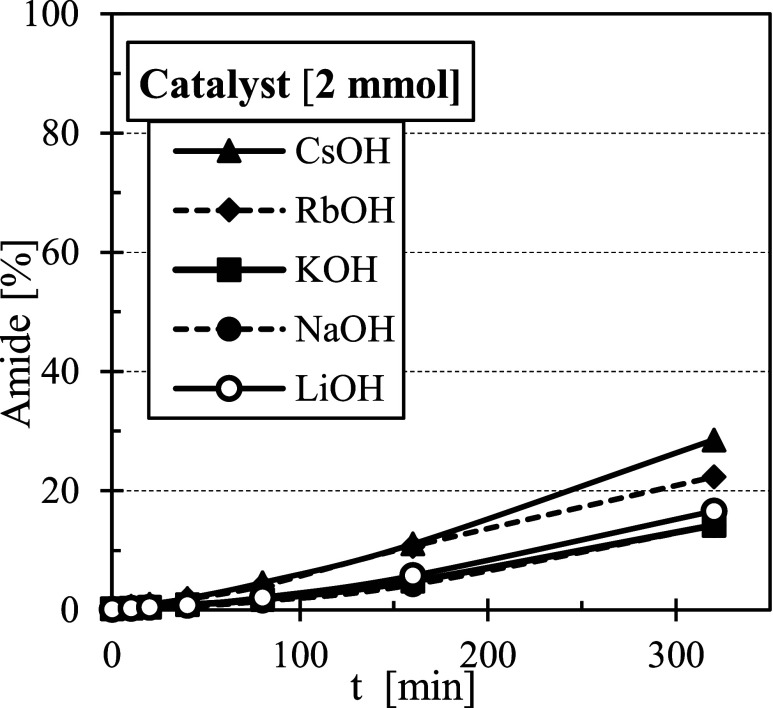
Effect of hydroxide catalysts on the reaction.

The catalytic effect can be explained based on the reaction mechanism, which is assumed to be similar to the aminolysis of esters described by Betts and Hammett.^25^ This reaction follows both uncatalyzed and base-catalyzed pathways

1 $${\mathrm{RCOOCH}}_{3}+{R^{\prime }\mathrm{NH}}_{2}\to {\mathrm{RCONHR}}^{\prime }+{\mathrm{CH}}_{3}\mathrm{OH}$$

2 $${\mathrm{RCOOCH}}_{3}+{\mathrm{RNH}}^{\prime -}\overset{{\mathrm{OH}}^{-}}{\to }{\mathrm{RCONHR}}^{\prime }+{\mathrm{CH}}_{3}O^{-}$$

In the uncatalyzed reaction (eq 1), the alkylamine reacts slowly with the ester, producing an amide and methanol. When a strong base is introduced as a catalyst, it deprotonates the alkylamine to generate an alkylamine anion (eq 2), which is a significantly stronger nucleophile. This anion reacts more rapidly with the ester, accelerating amide formation.^25^

To facilitate this deprotonation, a sufficiently strong base is required (eq 3)­

3 $${\mathrm{RNH}}_{2}+{\mathrm{CH}}_{3}O^{-}↔{\mathrm{RNH}}^{-}+{\mathrm{CH}}_{3}\mathrm{OH}$$

Consequently, catalysts with higher basicity exhibit greater efficiency. The basicity of metal hydroxides and methoxides increases down the group, with methoxides being more basic than their corresponding hydroxides. This trend follows the order

$$\mathrm{LiOH}<\mathrm{NaOH}<\mathrm{KOH}<\mathrm{RbOH}<\mathrm{CsOH}<CH_{3}\mathrm{OLi}<CH_{3}\mathrm{ONa}<CH_{3}\mathrm{OK}$$

which aligns with the observed catalytic efficiencies (Figure 3). Moreover, hydroxides may not be strong enough to effectively deprotonate the alkylamine, explaining their limited ability to accelerate the reaction compared to methoxides.^26^

### 3.3 Determination of Minimum Inhibitory Concentration

When evaluating the antimicrobial activity of synthesized compounds, the influence of both the functional group and alkyl chain length was explored. Regarding the functional group, no substantial differences were observed between the groups. However, a slight trend in decreasing antimicrobial efficiency can be observed in the following order: quaternary esters (compound 2A – 2D, Table 6), quaternary hydroxyamides (compound 3A – 3D, Table 7), and quaternary dihydroxyamides (compound 4A – 4D, Table 8). Although the differences are not significant, this pattern indicates that compounds with an ester group tend to show a modest advantage in antimicrobial activity compared with the hydroxyamide derivatives. Regarding alkyl chain length, QASs derived from myristic acid exhibited the highest antimicrobial activity, followed by palmitic acid derivatives, while lauric-acid–based compounds were the least effective. The most effective compounds were 2B (quaternary ester of myristic acid) and 3B (quaternary hydroxyamide of myristic acid), both exhibiting the strongest antimicrobial efficacy against *S. aureus*, *E. coli*, *P. aeruginosa*, and *A. brasiliensis*. The synthesized QASs showed the highest efficacy against the Gram-positive bacterium *S. aureus*, compared with the Gram-negative strains. This is a well-established fact, as Gram-negative bacteria possess a distinct cell wall structure that includes an outer membrane composed primarily of lipopolysaccharides, proteins, and phospholipids. The outer membrane acts as a barrier that QASs must pass through to reach the cytoplasmic membrane, where they disrupt its integrity.^2,7,11,14,27^

**Table 6:**
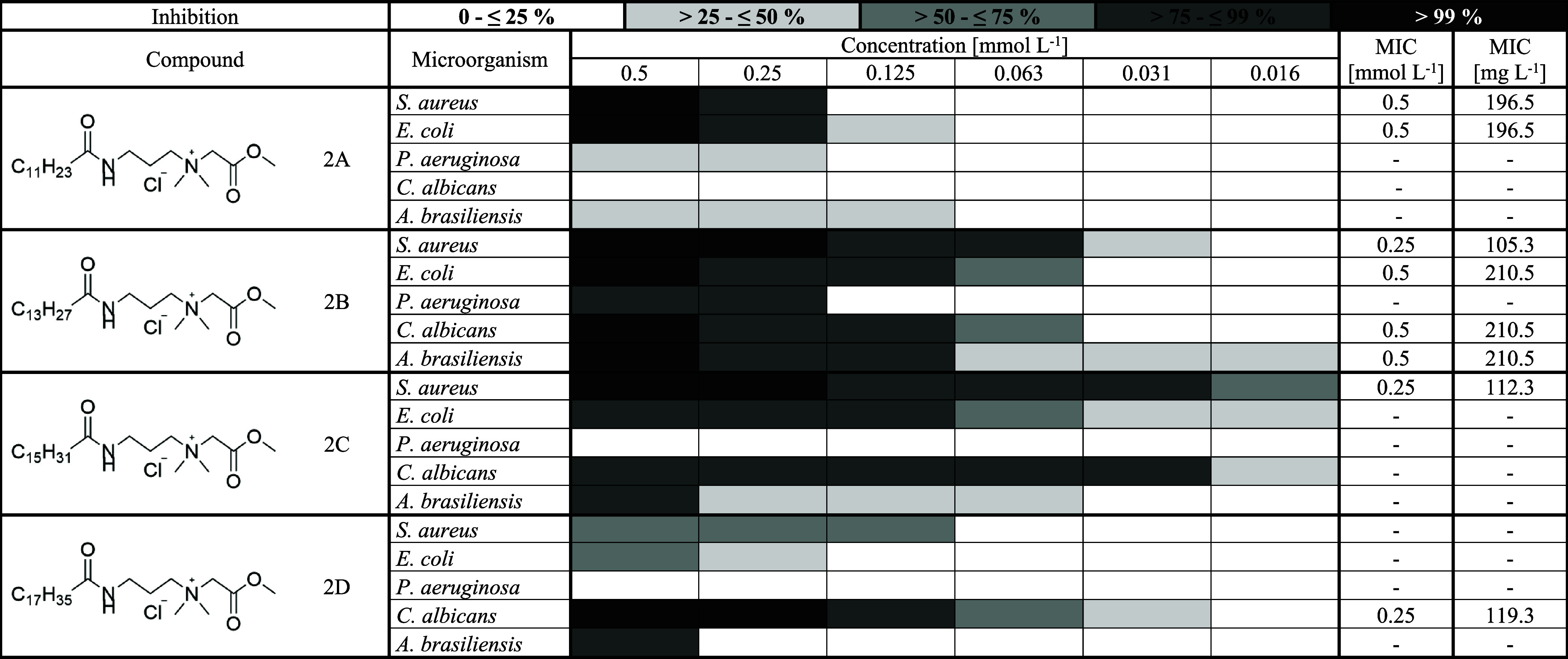
Antimicrobial Activity of Quaternary Esters^a^

a 2, Quaternary ester; A, lauric acid; B, myristic acid; C, palmitic acid; D, stearic acid; MIC, minimum inhibitory concentration [mmol L^–1^] and [mg L^–1^] at which 100% inhibition of the given microorganism is observed.

**Table 7:**
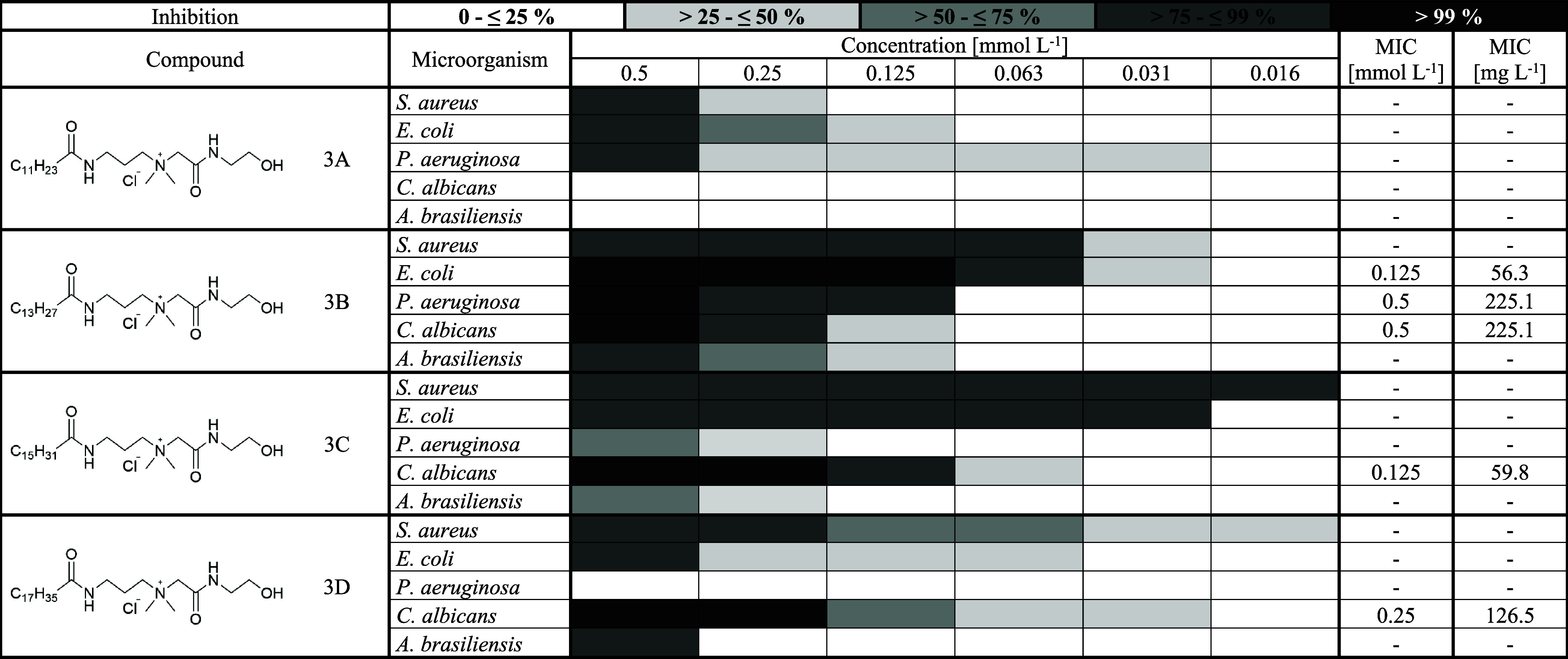
Antimicrobial Activity of Quaternary Hydroxyamides^a^

a 3, Quaternary hydrodroxyamide; A, lauric acid; B, myristic acid; C, palmitic acid; D, stearic acid; MIC, minimum inhibitory concentration [mmol L^–1^] and [mg L^–1^] at which 100% inhibition of the given microorganism is observed.

**Table 8:**
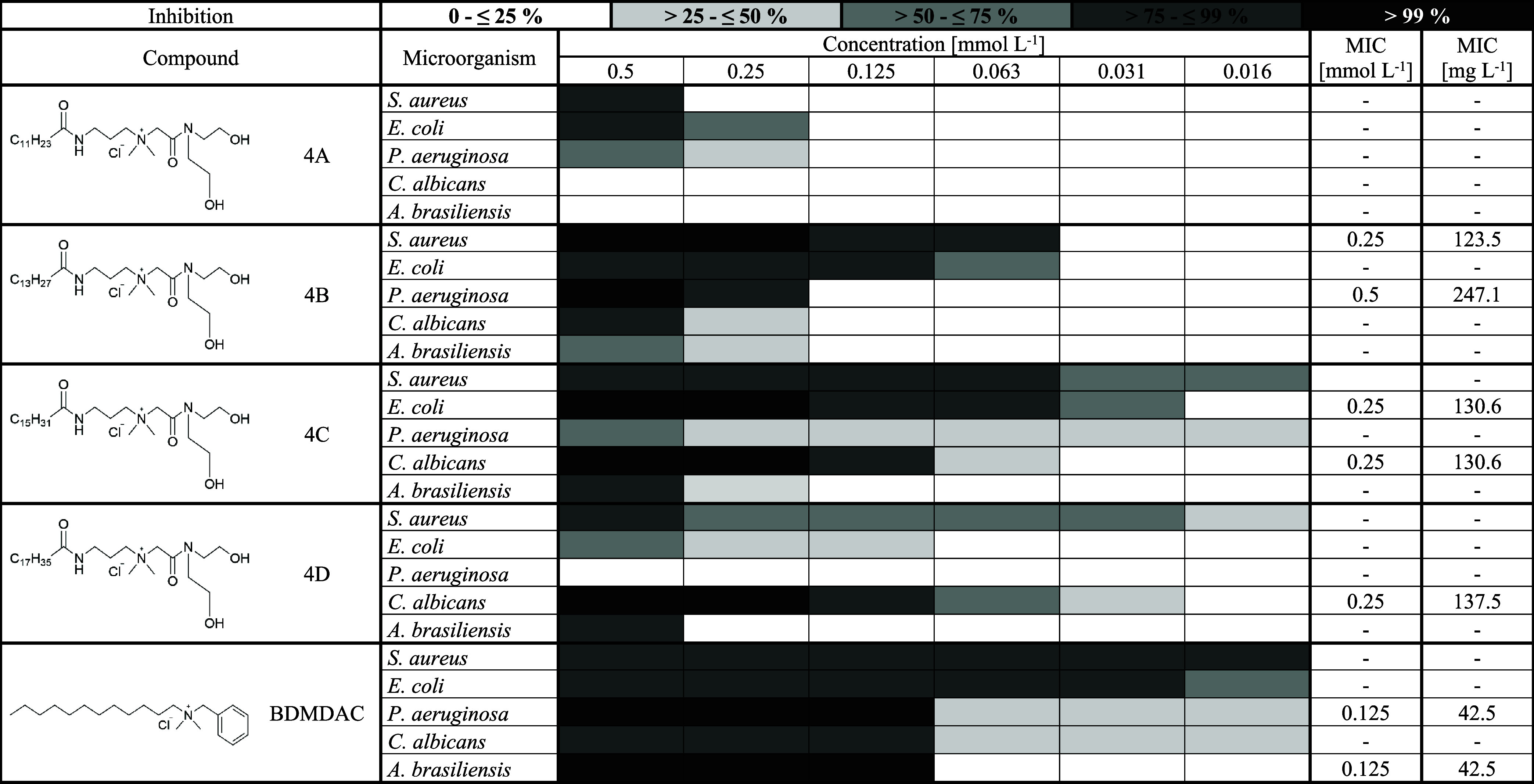
Antimicrobial Activity of Quaternary Dihydroxyamides and Benzyldimethyldodecylammonium Chloride^a^

a 4, Quaternary dihydrodroxyamide; A, lauric acid; B, myristic acid; C, palmitic acid; D, stearic acid; BDMDAC, beznyldimethyldodecylammonium chloride; MIC, minimum inhibitory concentration [mmol L^–1^] and [mg L^–1^] at which 100% inhibition of the given microorganism is observed.

When the relationship between MIC and CMC is considered, it becomes evident that both alkyl chain length and the balance between hydrophilic and hydrophobic parts influence antimicrobial activity. However, identifying a consistent trend is challenging, as multiple variables, such as microbial susceptibility and structural differences among QASs, may affect the observed antimicrobial behavior. In our study, we found that compounds with alkyl chains of 14 or fewer generally exhibited MIC values below their CMCs, indicating that monomers are likely responsible for the antimicrobial effect. In contrast, compounds with 16 carbon chains showed MIC values above the CMC, suggesting that aggregates or micellar forms may begin to play a more prominent role in the observed activity. These results are consistent with previous findings. Mikláš et al.^28^ reported that QASs with alkyl chains shorter than 16 carbon atoms had MIC values below their CMCs, while those with chains longer than 16 carbon atoms exhibited MICs above the CMC, indicating a shift from monomer-driven to aggregate-associated activity. Similarly, Joondan et al.^29^ observed that compounds with alkyl chains ranging from 10 to 14 carbon atoms had MICs below their CMCs, whereas those with 16 carbon chains or longer showed MICs above the CMC. These studies support the presence of an optimal hydrophobicity range, beyond which further increases in alkyl chain length promote micellization, potentially reducing the availability of biologically active monomers.

The most effective compounds against *S. aureus* were 2B, 2C, and 4B—derivatives of myristic and palmitic acid—all of which achieved 100% inhibition (MIC) at a concentration of 0.25 mmol L^–1^. Another promising compound was 3C, which, although it did not achieve full inhibition, consistently showed antimicrobial activity above 90% across all tested concentrations. On the other hand, the least effective were compounds 3A and 4A, derived from lauric acid. This was consistent with the findings of Kula.^2,14^

Although *E. coli* is a Gram-negative bacterium, the synthesized QASs showed good antimicrobial activity. The most effective were compounds 3B (MIC = 0.125 mmol L^–1^) and 4C (MIC = 0.25 mmol L^–1^). In contrast, the least effective were 2D and 4D, both derived from stearic acid. These findings are consistent with the literature, which reports that QASs with alkyl chain lengths of 14 and 16 carbon atoms exhibit the highest antimicrobial activity, whereas those with 18 carbon chains are significantly less effective.^8,14^ This decline in activity may be explained by the so-called *cutoff effect*. In a series of structurally related QASs, antimicrobial activity generally increases with alkyl chain length up to an optimal point, beyond which further elongation leads to a decrease in efficacy. This phenomenon can be attributed to several factors, including limited aqueous solubility, reduced membrane interaction, or molecular aggregation.^28,30^

*P. aeruginosa* was the most resilient of all tested microorganisms, which aligns with previous findings.^2^ One reason for its high resistance is the presence of an outer membrane, which serves as a protective barrier.^11^ Another contributing factor is the presence of efflux pumps in the *P. aeruginosa*, which actively transport QASs out of the cell, thereby lowering their intracellular concentration and reducing antimicrobial efficacy.^1,31^ Additionally, as reported by several publications, *P. aeruginosa* is able to metabolize QASs as a source of carbon and nitrogen.^32–34^ These combined resistance mechanisms may explain the reduced efficacy of the tested compounds. Despite this, compounds 3B and 4B achieved 100% inhibition at a concentration of 0.5 mmol L^–1^. In contrast, compounds 2C, 2D, 3D, and 4D exhibited no inhibitory activity against this bacterium.

When the antimicrobial activity of the synthesized QASs against *C. albicans* was evaluated, a trend of increasing efficacy with increasing alkyl chain length was observed. The most effective compounds were those derived from stearic acid (2D, 3D, and 4D), each exhibiting an MIC at 0.25 mmol L^–1^. In contrast, compounds based on lauric acid (2A, 3A, and 4A) showed no inhibitory activity at any tested concentration. A similar pattern was reported by Paluch et al., supporting the correlation between longer alkyl chains and enhanced antimicrobial activity. This effect is likely attributed to the ability of longer alkyl chains to more effectively interact with and destabilize the plasma membrane of *C. albicans*.^20^

In the case of *A. brasiliensis*, the synthesized QASs exhibited limited antifungal activity. The only compound that achieved 100% inhibition was 2B, with an MIC of 0.5 mmol L^–1^. In contrast, compounds 3A and 4A demonstrated no inhibitory effect under the tested conditions.

To evaluate the industrial potential of the synthesized QASs, the most promising compounds (2B and 3B) were compared with benzyldimethyldodecylammonium chloride (BDMDAC), a widely used disinfectant.^35,36^ While benzalkonium chloride (BAC) is more commonly used in the literature, typically as a mixture of quaternary ammonium compounds with varying alkyl chain lengths (e.g., C12, C14, and C16),^8,37–40^ in this study, the pure compound BDMDAC was used, which contains a C12 alkyl chain. Against *S. aureus*, compound 2B exhibited superior activity, achieving complete inhibition at 0.25 mmol L^–1^, while BDMDAC maintained >90% inhibition but did not reach full effectiveness. Compound 3B also demonstrated strong inhibition (>90%) at concentrations as low as 0.06 mmol L^–1^, indicating high potency. A similar trend was observed for *E. coli*, where compound 3B showed the strongest activity (MIC = 0.125 mmol L^–1^), followed by 2B (MIC = 0.5 mmol L^–1^). BDMDAC again did not reach full inhibition but maintained >90% inhibition up to 0.03 mmol L^–1^. For *C. albicans*, both synthesized compounds (2B and 3B) achieved complete inhibition at an MIC of 0.5 mmol L^–1^, exceeding the efficacy of BDMDAC under the same conditions. In contrast, BDMDAC was more effective against *P. aeruginosa* and *A. brasiliensis*, achieving full inhibition at 0.125 mmol L^–1^, whereas the synthesized compounds showed an MIC value of 0.5 mmol L^–1^. These results indicate that the synthesized QASs, particularly compounds 2B and 3B, are more effective than BDMDAC against three of the five tested microorganisms (*S. aureus*, *E. coli*, and *C. albicans*) and still show high activity against the remaining two (*P. aeruginosa* and *A. brasiliensis*). These findings support their potential as promising alternatives to commercial QAS-based disinfectants.

### 3.4 Determination of Biofilm Inhibition Activity

To compare the biofilm inhibition activities of synthesized QASs, we explored differences in both the functional group and the alkyl chain length. Across all tested compounds, those derived from palmitic acid showed the highest biofilm inhibition activity. When comparing different functional groups, the most effective were the quaternary hydroxyamides (compounds 3; Table 10), followed by quaternary esters (compounds 2; Table 9), while the quaternary dihydroxyamides (compounds 4; Table 11) showed the lowest activity overall. As for the alkyl chain length, the most effective compounds were those containing palmitic and then myristic acid (Tables 9–11). These findings are consistent with results reported by Kula et al.^2^ Regarding the tested microorganisms, QASs were most effective against *E. coli* and *S. aureus*. In comparison, their activity against *P. aeruginosa* was the lowest, likely because of several virulence factors that enhance biofilm resilience. In particular, Psl, Pel, and alginate form a dense extracellular matrix that protects the bacteria by limiting disinfectant and antibiotic penetration, making the biofilm much harder to disrupt or eliminate.^41^ Another explanation might be the presence of resistance mechanisms, such as efflux pumps, which remove the QAS from the bacteria.^42^

**Table 9:**
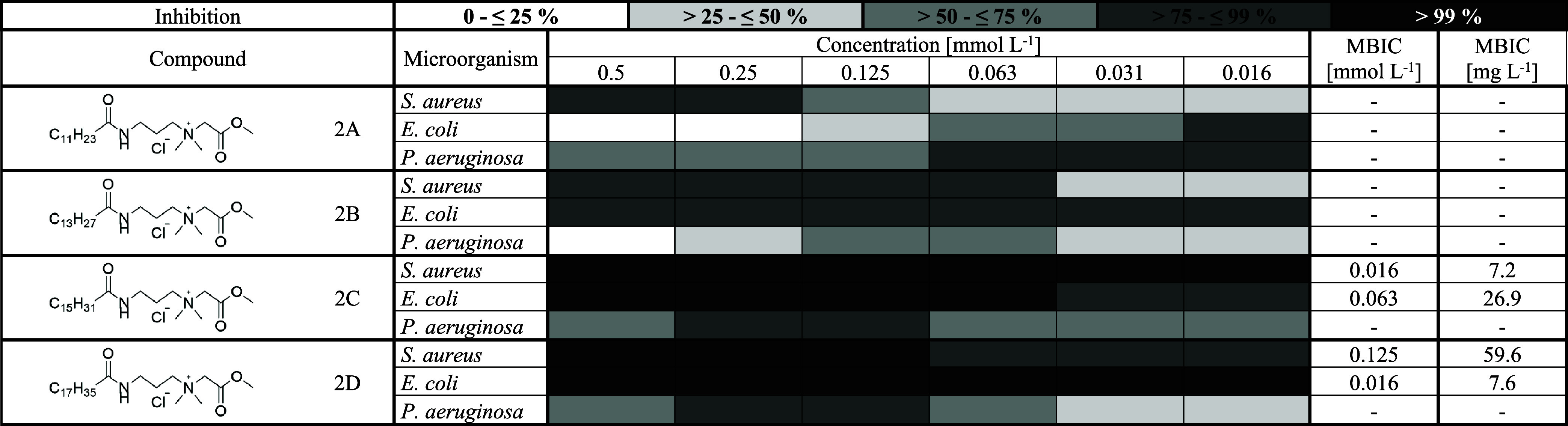
Biofilm Inhibition Activity of Quaternary Esters^a^

a 2, Quaternary ester; A, lauric acid; B, myristic acid; C, palmitic acid; D, stearic acid; MIBC, minimum inhibitory biofilm concentration [mmol L^–1^] and [mg L^–1^], at which 100% inhibition of biofilm formation is observed.

**Table 10:**
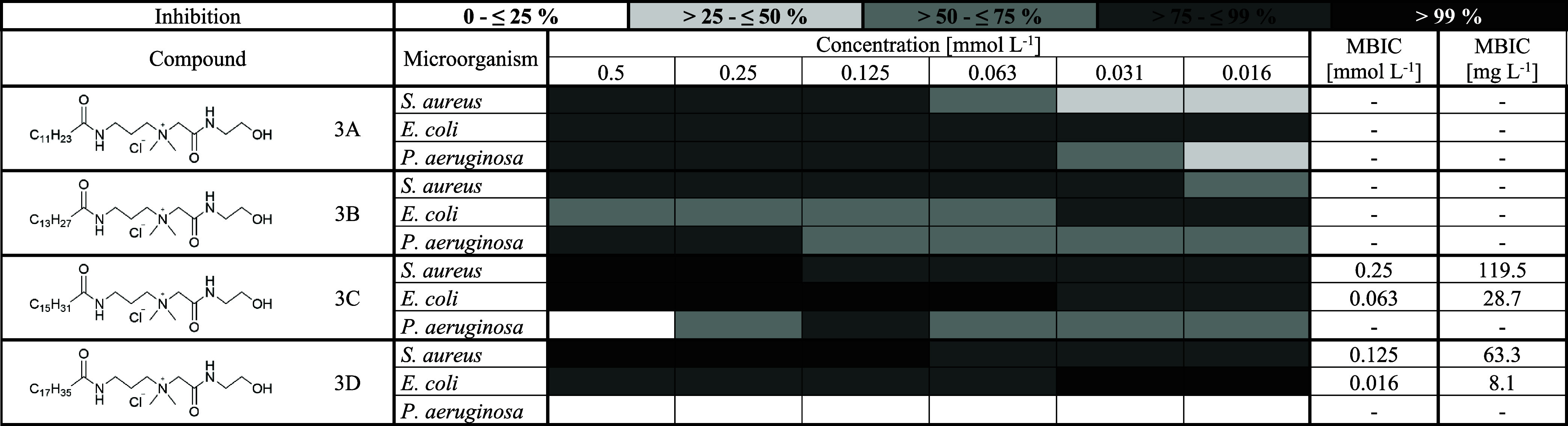
Biofilm Inhibition Activity of Quaternary Hydroxyamides^a^

a 3, Quaternary hydroxyamides; A, lauric acid; B, myristic acid; C, palmitic acid; D, stearic acid; MIBC, minimum inhibitory biofilm concentration [mmol L^–1^] and [mg L^–1^], at which 100% inhibition of biofilm formation is observed.

**Table 11:**
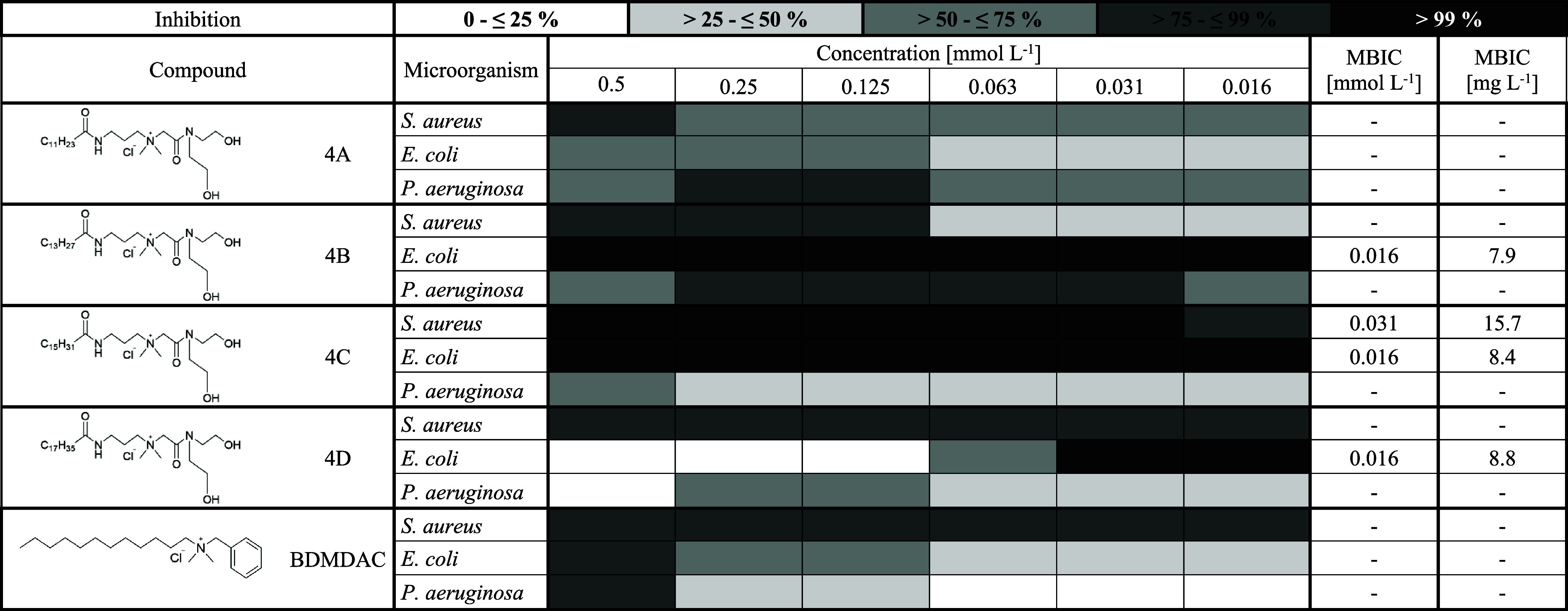
Biofilm Inhibition Activity of Quaternary Hydroxyamides and Benzyldimethyldodecylammonium Chloride^a^

a 4, Quaternary dihydroxyamides; A, lauric acid; B, myristic acid; C, palmitic acid; D, stearic acid; BDMDAC, benzyldimethyldodecylammonium chloride; MIBC, minimum inhibitory biofilm concentration [mmol L^–1^] and [mg L^–1^], at which 100% inhibition of biofilm formation is observed.

A more detailed look at the results against *S. aureus* confirmed that the palmitic acid derivatives were particularly effective. All three tested compounds (2C, 3C, and 4C) demonstrated strong biofilm inhibition activity; however, compound 2C (a quaternary ester of palmitic acid) stood out with the lowest minimum biofilm inhibitory concentration (MBIC), reaching values as low as 0.016 mmol L^–1^, followed by 4C (MBIC 0.031 mmol L^–1^) and then 3C (MBIC 0.25 mmol L^–1^). On the other hand, compounds derived from lauric acid (2A, 3A, and 4A) showed the lowest biofilm inhibition activity. These findings suggest that both the structure of the functional group and the length of the alkyl chain play a significant role in determining biofilm inhibition activity and that esters of palmitic acid may be especially promising candidates for further development.

As for the biofilm inhibition activity against *E. coli*, the tested compounds showed the highest overall efficacy against this bacterium. The most effective compounds were 4B (quaternary dihydroxyamide of myristic acid), 4C (quaternary dihydroxyamide of palmitic acid), and 2D (quaternary ester of stearic acid), all reaching MBIC values of 0.016 mmol L^–1^. Interestingly, for certain compounds (2A, 3B, 3D, and 4D), biofilm inhibition activity was greater at lower concentrations and declined as the concentration increased. Notably, compounds 3D and 4D reached MBIC at 0.016 mmol L^–1^, while at the same time, compound 4D showed no inhibition from 0.125 mmol L^–1^. This may be explained by the paradoxical effect, where higher concentrations of antimicrobial agents become less effective. One possible explanation is that sublethal QAS levels can activate quorum sensing and stress responses in *E. coli*, promoting biofilm formation as a defense mechanism. At higher doses, rapid killing may leave behind a resistant subpopulation that continues to form biofilms enriched with protective features, making inhibition less effective.^43^ Another possible explanation is that some subinhibitory concentrations of antibiotics can stimulate autolysis in certain bacteria, leading to the release of DNA, which can then integrate into the biofilm matrix. Similarly, QAS at concentrations below the lethal level might cause partial cell lysis. The released extracellular DNA (eDNA) and debris could facilitate attachment of the surviving cells, resulting in a more persistent residual biofilm. In contrast, a lower QAS dose may avoid triggering such lysis, allowing cells to remain planktonic and easier to remove.^44^ When discussing QAS, it is also important to consider whether the applied concentration is above or below the critical micelle concentration (CMC). Once the CMC is exceeded, QAS molecules begin to form micelles, which reduces the amount of active monomeric QAS available to interact with bacterial cells. This effect is particularly relevant for QAS with long alkyl chains and may lead to lower concentrations being more effective for biofilm inhibition.^2,28^ This might explain the behavior of the compounds 3D and 4D, where the MBIC was below the CMC (CMC was 5.69·10^–5^ mol L^–1^ for compound 3D and 3.69·10^–5^ mol L^–1^ for compound 4D), and inhibition decreased as the concentration approached or exceeded the CMC.

As for *P. aeruginosa*, this bacterium proved to be the most resilient among the tested strains, which might be due to virulence factors and efflux pumps, as mentioned before.^41,42^ None of the tested QASs achieved complete biofilm inhibition. In this case, the inhibitory effect decreased with increasing alkyl chain length, with the most effective compounds being those derived from lauric acid, specifically compound 2A, which showed 75–99% inhibition at concentrations between 0.063 and 0.016 mmol·L^–1^. This is likely due to limited diffusion or stronger adsorption to organic materials in QAS with longer alkyl chains. In contrast to biofilm inhibition, the most effective antimicrobial activity against planktonic cells was observed with compounds containing a 14-carbon alkyl chain, particularly compounds 3B and 4B, which reached MIC at 0.5 mmol L^–1^. The resistance of *P. aeruginosa* biofilms to QAS appears to be primarily caused by the protective biofilm matrix rather than by an intrinsic resistance mechanism. As demonstrated by Campanac et al., removal of the matrix restored the sensitivity of *P. aeruginosa* to QAS, indicating that reduced penetration and matrix-associated shielding are the main factors limiting efficacy.^40^

For compound 2A (quaternary ester of lauric acid), we observed a similar paradoxical effect as seen previously with *E. coli*, where lower concentrations resulted in greater biofilm inhibition activity. A notable finding was that compounds 2B, 2C, 2D, 3C, 4A, 4B, and 4D exhibited an inverted U-shaped pattern of biofilm inhibition, with maximum efficacy at intermediate concentrations, while both lower and higher concentrations showed reduced activity. A possible explanation for this nonmonotonic response can be drawn from Alsamhary, who demonstrated that subinhibitory concentrations of QACs can activate quorum-sensing pathways in *P. aeruginosa*, thereby promoting biofilm formation and virulence.^16^ On the other end of the concentration spectrum, very high concentrations may lead to micelle formation or limited penetration due to compound aggregation, as noted in other studies. For example, Kula et al. report that at concentrations below the CMC, some QACs may form premicellar aggregates, which can reduce molecular mobility and potentially diminish antimicrobial activity, especially in complex environments such as biofilms.^2^ Thus, at moderate concentrations, QACs may most effectively interfere with biofilm development. This allows them to avoid triggering protective stress responses. At the same time, it helps prevent the loss of efficacy caused by physicochemical inactivation. Together, these factors may explain the observed peak in the biofilm inhibition activity. While several possible explanations exist for these findings, the precise mechanisms remain unclear and require further investigation.

Lastly, we compared our synthesized QASs with benzyldimethyldodecylammonium chloride (BDMDAC), a compound commonly used in industry. Our QASs consistently showed significantly higher biofilm inhibition potential. BDMDAC never achieved 100% inhibition at any tested concentration, whereas several of our compounds reached the minimum biofilm inhibitory concentration (MBIC) multiple times. For instance, against *S. aureus*, BDMDAC achieved over 75% inhibition at all tested concentrations, but compound 2C reached MBIC at just 0.016 mmol·L^–1^, and compound 4C reached MBIC at 0.031 mmol·L^–1^. In the case of *E. coli*, BDMDAC showed over 75% inhibition only at 0.5 mmol·L^–1^, while compounds 2D, 4B, and 4C all reached MBIC at 0.016 mmol·L^–1^. Finally, for *P. aeruginosa*, BDMDAC exceeded 75% inhibition at 0.5 mmol·L^–1^, whereas the most effective compound, 2A, achieved comparable inhibition already at 0.016 mmol·L^–1^. These results clearly suggest that our synthesized QASs have biofilm inhibition activity considerably higher than that of BDMDAC. This highlights their potential for use as disinfectants in hospitals and other settings where effective biofilm control is essential.

## 4 Conclusions

In this work, we successfully synthesized 12 novel quaternary ammonium salts (QASs). The first step of the synthesis, involving amide formation, was optimized by evaluating various catalysts, with CH_3_OK proving to be the most effective. The antimicrobial and biofilm inhibition properties of the synthesized QASs were then evaluated, and their efficacy was compared with the commonly used disinfectant BDMDAC. Among the synthesized compounds, those derived from myristic and palmitic acids consistently showed the highest antimicrobial and biofilm inhibition efficacy, often outperforming BDMDAC across multiple bacterial strains. While lauric acid–based QASs were generally less effective, the quaternary ester of lauric acid (2A) stood out for its strong biofilm inhibition activity against *P. aeruginosa*. Interestingly, quaternary ester of lauric acid (2A), quaternary hydroxyamide of myristic acid (3B), quaternary hydroxyamide of stearic acid (3D), and quaternary dihydroxyamide of stearic acid (4D) demonstrated greater biofilm inhibition activity against *E. coli* at lower concentrations, which could help reduce chemical exposure in the applied settings. Overall, these findings suggest that the newly synthesized QASs—particularly those based on myristic and palmitic acids—are strong candidates for further development as effective disinfectants, especially in settings where biofilm control is a significant challenge.
